# Progress in the application of organoids to breast cancer research

**DOI:** 10.1111/jcmm.15216

**Published:** 2020-04-13

**Authors:** Liping Yang, Baoer Liu, Haodong Chen, Rui Gao, Kanghua Huang, Qiuyi Guo, Feng Li, Weicai Chen, Jinsong He

**Affiliations:** ^1^ Department of Breast Surgery Peking University Shenzhen Hospital Shenzhen China; ^2^ Department of Breast Surgery Shenzhen Second People′s Hospital the First Affiliated Hospital of Shenzhen University Shenzhen China

**Keywords:** breast cancer, organoid, studying model

## Abstract

Breast cancer is the most common cancer diagnosed in women. Breast cancer research is currently based mainly on animal models and traditional cell culture. However, the inherent species gap between humans and animals, as well as differences in organization between organs and cells, limits research advances. The breast cancer organoid can reproduce many of the key features of human breast cancer, thereby providing a new platform for investigating the mechanisms underlying the development, progression, metastasis and drug resistance of breast cancer. The application of organoid technology can also promote drug discovery and the design of individualized treatment strategies. Here, we discuss the latest advances in the use of organoid technology for breast cancer research.

## 1 INTRODUCTION

Breast cancer (BC) is the most common invasive cancer in women.^1^ It affects approximately 12% of women worldwide,^2^ and in 2012, it accounted for 25.2% of cancers diagnosed in women, making it the most common female cancer.^3^ BC is usually treated with surgery, which may be followed by chemotherapy, radiation therapy or both; a multidisciplinary approach is preferable.^4^ Current research on BC focuses on understanding its genesis and development, and on elucidating metastasis and drug resistance mechanisms. The design of individualized treatment strategies for patients with BC is an important topic. Research on tumour biological behaviour has remained at the level of traditional tumour cell lines and animal models despite the gap between different species and between in vitro and in vivo environments. This has become a stumbling block in the application of increasingly sophisticated high‐throughput genomics to clinical research.

In 1907, Henry Van and Peters Wilson demonstrated that mechanically isolated sponge cells can regroup and self‐organize to produce a whole organism.^5^ The subsequent development of cell biology revealed the existence of stem cells, which can differentiate into various types of cells.^6^ The emergence of stem cell biology demonstrated the potential of stem cells for organogenesis in vivo. Stem cells can form teratomas or embryoids, and differentiated cells organize into different structures comparable to those found in multiple tissue types.^6^ The differentiation and transformation of stem cells from a two‐dimensional (2D) to a three‐dimensional (3D) culture system, which enables the development of complex 3D organ structures, led to the emergence of the organoid field.^6^ Since 1987, different 3D culture systems have been developed, and different types of stem cells are used for producing organoids mimicking many organs. An organoid is an in vitro model derived from stem cells. After 3D system, organoids contain a variety of cell types and can self‐organize in a manner similar to their in vivo behaviour by proliferation and differentiation. As a result, they form structures that retain the original organ identity in vivo.^7^ The organoid technique first employed organs with abundant epithelial structures, such as the stomach,^8^ small intestine,^9^ colorectum,^10^ pancreas,^11^ breast^12^, ^13^ and prostate.^14^ Recently, organoids have become a new trend for studying the evolution of tumours and evaluating the efficacy and toxicity of drugs. This is because organoids have unique characteristics that allow them to reveal most of the tumour properties at the in vitro level. Organoids can be used for exploring the role of cancer stem cells and tumour metastasis mechanisms, as well as for studying the biological characteristics of tumour cells accurately.^15^ This review focuses on the application of organoids to BC research.

## 2 THE ORIGIN OF THE MAMMARY ORGANOID

From the 3D culture models of normal mammary epithelial cells to the establishment of 3D culture system supporting the growth of human breast primary epithelial cells, the culture of mammary organoid has also experienced gradual development.^13^, ^16^, ^17^ The latter facilitates the growth of morphologically complex and hormone‐sensitive mammary tissues. The primary human epithelial cells were self‐organized and showed complex vessels and lobular morphologies in the tissue. The ability to culture hormone‐sensitive human mammary tissue in hydrogels with defined components will promote the development of human mammary gland (HMG)‐based research, which has potential implications for understanding the biology of mammary cancer.^17^ In 2017, Qu et al described a method for generating human mammary‐like cells from induced pluripotent stem cells (iPSCs). Human iPSC (hiPSC)‐derived mammary‐like organoids can be used for establishing in vitro models to elucidate the precise effects of various factors on breast cell transformation and BC development, as well as for personalized bioengineering of breast tissue.^18^

### 2.1 Developments in breast stem cell research

The leucine‐rich repeat‐containing G protein‐coupled receptor 5 (Lgr5) is widely known as a stem cell marker in multiple mammalian tissues, such as the stomach, intestine and skin.^19^, ^20^, ^21^, ^22^ Lgr5, the receptor for R‐spondin proteins, is a Wnt‐mediated signal transduction agonist through the β‐catenin/TCF pathway.^21^, ^22^ Visser et al, Rios et al and Plaks et al suggested Lgr5 as a potential breast stem cell marker.^23^, ^24^, ^25^ In addition, fat pad transplantation tests showed differences between Lgr5+ cells and Lgr5‐ cells. Werb et al indicated that Lgr5+, but not Lgr5‐ cells, could form whole mammary glands.^24^ However, Rios et al suggested that both Lgr5+ and Lgr5− cells show regeneration potential in transplantation experiments.^25^ Wang et al reported that Lgr5‐ rather than Lgr5+ cells form colonies in 3D culture.^26^ Zhang et al isolated Lgr5+ cells from the mammary glands of Lgr5‐lacZ mice and established breast organoids. The colonies from a single Lgr5+ cell spontaneously form a ductal structure surrounded by basal cells. The lumen cells are arranged in a manner resembling the normal ductal structure of the breast. Lgr5+ cell‐derived organisms are sustainable during long‐term passage; however, although Lgr5‐ cells expand into primary colonies, the efficacy of colony formation decreases immediately after passage. In addition, reproductive hormones induce epithelial cell proliferation, leading to a significant increase in lumen diameter, accompanied by squamous cell differentiation. Taken together, these findings support the use of mammary Lgr5+ cells as legitimate mammary stem cells.^27^

### 2.2 Culture of mammary gland organoids in vitro

Induced pluripotent stem cells can be produced directly from terminally differentiated cells.^28^ This bypasses the need for embryos, and iPSCs from different individuals can be used to model personalized or patient‐specific diseases. HiPSCs can produce a variety of cell types, such as neurons, cardiomyocytes and hepatocytes.^29^ Qu et al described a method to generate human mammary‐like cells even a mammary gland from iPSCs using a suspension sphere culture system. This method is based on the use of non‐neural ectoderm progenitors and a mixed gel floating 3D culture system, which is used to simulate the extracellular matrix (ECM) of mammary gland differentiation. The hiPSC‐derived mammary‐like organoids can be used to construct in vitro models to examine the effects of various factors on breast cell transformation, breast cancer development and breast tissue individualized bioengineering.^30^

### 2.3 Organoids are an important tool for breast cancer research

The most commonly used models for studying BC are cell lines and patient‐derived xenografts (PDX).^31^, ^32^ Both model systems have considerable drawbacks, although they have contributed greatly to translational BC research.^33^ Tumour cell lines acquire mutations during the culture process, which cannot faithfully simulate the original characteristics of the tumour. In addition, cell culture cannot simulate the interaction between tumour cells and other stromal cells in vivo, as cultured cells are single and lack the hierarchy of different cell types.^34^ PDX have a wide range of applications; however, they cannot fully reflect the genetic characteristics and heterogeneity of human tumours.^35^, ^36^, ^37^ PDX cannot be used to study the process of tumorigenesis, and tumour xenotransplantation has many limitations, such as time‐consuming, laborious, long culture cycle, inefficient and difficult for high‐throughput drug screening work.^38^ Organoid culture can maintain the original genotype and biological characteristics of the tumour. It has other advantages such as stable passage, relatively simple operation and short culture cycle. Organoid culture technology is very helpful for studying the differentiation of cancer stem cells into different types of tumour cells, revealing the causes of tumour heterogeneity, evolution and metastasis, and for evaluating the efficacy of drugs.

Patient‐derived tumour organoids (PDTOs) are pre‐clinical models for tumour propagation in vitro. PDTOs provide an excellent platform for the study of tumour progression, invasion and drug responses, as they reflect the cellular heterogeneity present in the primary neoplasm.^39^, ^40^ However, organoids are limited by the lack of innervation, blood vessels and immune cells.^41^ We compare the differences between the three models in Table 1. Currently, efforts are being made to overcome these limitations. One example is the application of co‐culture techniques for organoids and mammospheres. Co‐cultures of Vδ2 + T lymphocytes and organoids derived from primary human mammary epithelial cells have been successful, and these T lymphocytes can effectively eradicate triple‐negative breast cancer (TNBC) cells.^42^ These findings suggest that T lymphocytes from healthy blood donors can be amplified and activated by organoids and subsequently used to treat patients, as well as offering the possibility of in vitro cytotoxicity tests of T lymphocytes from healthy blood donors to tumours from patients. The results of this study support the organoid as an effective model for the study of tumour progression, invasion and drug responses.

**TABLE 1:** The difference between cell lines, PDX and PDTOs

	Cell lines	PDX	PDTOs
Origin	Advanced tumour	Advanced tumour	Patient
Physiologic representation	Limited	Physiologic	Semi‐physiogic
Vascularization and immune system	No	Yes	No
Manipulability	Excellent	Limited	Good, but may have experimental variability
Biobanking	Yes	Yes, but only at the cellular level	Yes
Genome editing	Yes	Yes, but may require generation of embryonic stem cells	Yes
Modelling editing	Poor	Yes, but often confounded by complex tissue environment	Suitable for study of cell‐cell communication, morphogenesis; reduced complexity
Derivative efficiency	Low	Low	High
Reflect heterogeneity of primary tumours	No	Partially yes	Yes
Be widely used in individualized treatment	No	No	Yes
Fully capture the BC spectrum	No	No	Yes
Apply to high‐throughput drug screening	No	No	Yes

### 2.4 Gene editing of tumour organoids and establishment of tumour organoid animal models

Early studies showed that breast adenocarcinomas in BRCA‐related hereditary breast cancer K14cre; Brca1F/F; p53F/F (KB1P), K14cre; Brca1F/F; p53F/F; Mdr1a/b‐/‐(KB1PM), and K14cre; Brca2F/F; p53F/F (KB2P) mouse models summarize the key features of human diseases including morphologic, expression of basal labels, genomic instability and hypersensitivity to targeted DNA therapy.^43^, ^44^, ^45^ Duarte et al used CRISPR‐Cas9 gene editing to cultivate PARPi‐naive BRCA1‐deficient mammary tumour organoids with the Trp53bp1 mutation and detected the response to olaparib therapy in vivo. Compared with control tumour organoids, which were highly sensitive, transplanted tumour organoids with TRP53BP1 targeting tissue‐KB1PM7N.1 showed a limited response to olaparib. The results indicated that the deletion of 53BP1 produced a substantial selective advantage in KB1PM tumour cells, even without PARPi treatment. Moreover, Trp53bp1 frameshift mutations were further enriched after olaparib treatment. Immunohistochemical analysis confirmed the deletion of 53BP1‐positive tumour cells. This is consistent with the known role of 53BP1 deletion in PARPi resistance.^46^ These results indicate that the CRISPR/CAS9 system can effectively modify GEMM‐derived mammary tumour organoids to target genes of interest.

### 2.5 Research on the mechanism of breast cancer

Connexin 43 (Cx43) gap junctions are generally down‐regulated in human mammary cancer tissues compared with the non‐neoplastic mammary gland tissue surrounding primary tumours.^47^ In addition, both Cx26 and Cx43 are down‐regulated in many breast cancer cell lines, indicating that gap junctions play a role in maintaining cell differentiation and preventing transformation.^48^, ^49^, ^50^, ^51^ Conversely, when connexin is overexpressed in cancer cells, tumour growth slows down, and the cells regain the ability to form at least some differentiated structures.^52^ Mice lacking Cx32 are highly sensitive to liver and lung tumours, and connexins undoubtedly have tumour‐suppressive properties.^53^, ^54^, ^55^, ^56^ Early studies showed that retroviral delivery of Cx26 or Cx43 to MDA‐MB‐231 cells in 2D culture did not significantly increase gap junctional intercellular communication (GJIC) or inhibit growth, whereas it caused in vivo growth inhibition.^57^ McLachlan et al investigated connexin tumour inhibition patterns in 3D organs. Compared with the MDA‐MB‐231 cells in most control groups, the cells expressing Cx26 or Cx43 grew as spherical organs resembling the acinar growth of normal mammary epithelial cells. In addition, connexin expression allowed partial redifferentiation of MDA‐MB‐231 organoid growth and reduced anchor‐dependent growth, although it did not significantly promote the formation of gap junction plaques or rescue gap junction‐mediated dye transfer. This discovery provides a model for investigating the independent inhibitory effect of connexin GJIC on tumours.^58^ The expression of Cx26 or Cx43 partially restores epithelial to mesenchymal transition associated with cell transformation.

Nguyen‐Ngoc et al used Matrigel to model the normal mammary epithelium microenvironment, and type I collagen to mimic the stromal matrix of invasive mammary carcinomas. These authors showed that changes in ECM‐induced signals could initiate invasion and local dissemination.^59^ Organoids derived from breast carcinoma cells are used to understand the potential mechanisms underlying the invasiveness of tumour cells and metastasis.^60^, ^61^ Cheung et al found that in the major subtypes of human breast carcinoma, specialized cancer cells that express basal epithelial genes such as cytokeratin‐14 and p63 promote collective invasion. Their results suggested that heterotypic interactions between epithelial subgroups are the basis of collective invasion.^39^

### 2.6 Research on the treatment of breast cancer

Sachs et al generated 12 BC organoid lines from needle biopsies of 13 patients with metastatic BC. Tamoxifen elicited differential responses, as one patient was responsive, one was non‐responsive and the remainder were undetermined. In vitro responses of BC organoids to tamoxifen matched those of the respective patients, indicating the potential use of BC organoids as predictive in vitro surrogates for BC in vivo.^62^ Walsh et al prepared organoids from six different samples of primary mammary cancer tumours to predict treatment response. They used optical metabolic imaging to quantify the fluorescence intensity and half‐life of NADH and FAD, thereby measuring the sensitivity to treatment. Compared with xenograft‐like organs, PDTOs differ between patients and among breast cancer subtypes, showing significant heterogeneity in primary tumours. Walsh et al showed that optical metabolic imaging of organoids derived from primary tumours is useful for the early prediction of the response to therapy and for measuring antitumour drug responses in human‐tumour derived organoids, which allows testing the efficacy of a panel of drugs for selecting optimal drug combinations.^63^

Because of the innate potential of progenitor cells to differentiate into various cell types, bone marrow‐derived mesenchymal stromal cells (MSCs) are increasingly used for regeneration of damaged tissues in clinic.^64^, ^65^, ^66^, ^67^, ^68^ Studies support the value of cellular and gene therapy strategies for the implantation of genetically engineered autologous MSCs into the matrix, which serve as a neo‐organoid for therapeutic protein delivery. These techniques are not only feasible, but also promising for mammary cancer treatment.^69^ Eliopoulos et al have been suggested that MSCs genetically engineered to express interleukin‐12 (IL‐12) are embedded in the matrix when delivered subcutaneously to autologous/homologous hosts and can act as an anticancer neo‐organoid. They produce replication‐free IL‐12‐containing retroparticles and replication‐free control retroparticles, and breed IL‐12 MSCs and control MSC neo‐organoids. Researchers observed that primary murine MSCs secreting murine IL‐12 by retroviral engineering interfere with the growth of 4T1 breast cancer cells in vivo. When these cells were embedded in the matrix, substantial anticancer effects were achieved. Plasma levels of IL‐12 and IFN‐γ were increased in mice receiving IL‐12 MSC‐containing neo‐organoids. Histopathological analysis showed that implantation of IL‐12 MSCs into 4T1 cells reduced tumour cells and resulted in the appearance of necrotic tumour islets and necrotic capillaries, which have anti‐angiogenesis effects. Researchers also found that the anticancer effect of IL‐12 MSCs was immune‐mediated, as it did not occur in immunodeficient mice. Therefore, researchers confirmed the feasibility of gene‐enhanced MSCs for cancer treatment in a cell‐based neo‐organoid pathway.^70^

### 2.7 Challenges and future directions

The main challenge of pre‐clinical cancer research is still to establish a model that can summarize the patient's situation as close as possible and retain the intra‐tumour heterogeneity and the tumour environment. PDTOs can be used for high‐throughput drug screening and selection of effective drugs or drug combinations and to verify the efficacy of these selected drugs.^18^ This pre‐clinical model can reflect the response of anticancer therapy and give tailored treatments for patients. For example, the organoids growing from the cancer focus during biopsy can guide the individualized treatment plan of patients who need neoadjuvant chemotherapy and palliative treatment without any additional inconvenience to the patients, while the organoids growing from the healthy tissues can provide general information about the drug toxicity.^71^ In addition, organoids grown from the patient's liver tissue can be tested to determine its hepatotoxicity, or potential therapies for cardiotoxicity can be obtained from heart cells.^72^

In order to develop PDTO as a clinical test that can guide the treatment of prospective cancer patients, initial clinical studies should be designed to measure the sensitivity and specificity of empirical PDTO to a large number of the same patients receiving the identical drug treatment. At the same time, other potential predictive biomarkers of therapeutic response, such as chemosensitivity gene expression characteristics, can be evaluated in a similar way. If PDTO empirical test can reflect the response of a large number of patients, PDTO should be further developed as a laboratory test and properly evaluated in clinical trials. Currently, PDTO can be used to select second‐line or adjuvant therapy, because the time required to generate and test PDTO is about 4‐6 weeks. Reducing PDTO development and drug testing to one week requires innovation, but it can also be evaluated as a prospective test for cancer patients.^73^

Optical metabolic imaging (OMI) provides a non‐invasive method for cell metabolism measurement by using the ratio of NADH and FAD. The redox ratio can provide reliable metabolic readout, so that the technology is superior to those based on single‐molecule fluorescence.^74^ Imaging technology has many advantages. High resolution makes it possible to track single cells, which helps to identify resistant populations within organoids.^75^, ^76^ Detection of drug‐resistant populations can lead to a more appropriate combination of drugs, thus avoiding patients receiving ineffective treatment.^77^ In addition, OMI significantly reduced the time required to determine potential therapeutic effects, from three weeks of xenotransplantation studies to just 72 hours.^77^

However, many laboratories are still faced with the challenge of successfully cultivating organoids from patient samples. At present, the majority of PDTO specimens are still from surgical resection tissues. Because of the lack of cells in the biopsy specimen, it is often difficult to develop a qualified organoid model, which makes the patients who need neoadjuvant chemotherapy or palliative chemotherapy benefit little. Multipoint biopsy may be a solution. Sachs et al have increased the success rate of BC organoid establishment to more than 80% with optimized BC organoid culture medium.^62^ Compared with previously established human organoid protocols,^10^, ^78^ they emphasized that (a) the addition of neuregulin 1 allowed the efficient generation of BC organoids and their long‐term expansion; (b) the addition of specific Rho‐associated coiled‐coil containing protein kinase (ROCK) inhibitor Y‐27632 could improve the culture conditions; (c) the addition of Wnt‐3A did not significantly improve the culture conditions; (d) high concentration of epidermal growth factor (EGF) increased proliferation, but caused BC organoids to sink gradually and lose their 3D organization; and (e) high concentration of SB202190 decreased organoid establishment efficiency.^62^, ^79^, ^80^ Different teams have been using different protocols and different mixtures of inhibitors and growth factors to find the most suitable medium for their growth.^30^, ^62^, ^81^, ^82^ This may affect the results obtained in different laboratories and hinder data comparison. In addition, the composition of Matrigel varies from batch to batch, which leads to the difference between experiments.^13^, ^83^ This highlights the need for a standard protocol that must reliably integrate the best conditions for breast cancer organoids.

## 3 DISCUSSION

For decades, BC pre‐clinical research has relied on different cell lines as in vitro representations of a heterogeneous disease affecting millions of patients. Although high‐throughput screening is possible, BC cell lines do not capture the spectrum of BC completely, and there is little clinical relevance for individual patients. As the other pillar of pre‐clinical BC research, PDX models capture tumour heterogeneity, whereas they do not allow traditionally high‐throughput screening. To overcome these issues, several groups directly treat mice in vivo or PDX‐derived cultures in vitro. Although promising, the latter does not allow prolonged passage in vitro, and both methods are limited by inefficient PDX production. Organoid cultures represent a related biological tool that eliminates the gap between in vitro cultures of 2D animals and animal experiments. In addition, they provide an alternative to animal experiments and can help predict human risks associated with individual therapy in breast cancer. Tumour organoids can be used to study the evolution of tumours, evaluate the efficacy and toxicity of drugs, explore the role of cancer stem cells and the mechanism of tumour metastasis and accurately study the biological characteristics of cancer cells.

Although organoids have broad application prospects, they lack mesenchymal cell support, nerve innervation and vascular support; therefore, there is a considerable gap between organoids and real organs. The emergence of a new organoid culture model allows co‐culture of epithelial organoids and stromal cells, and further studies of the interaction between tumours and stromal cells can be carried out using organoid culture. Advances in the co‐culture of tumour‐like organoids with nerve and vascular tissues are also bridging the gap in organoid culture. In spite of these limitations can be overcome in theory, it is difficult to model the immune microenvironment around tumour. Tumour immune system is a complex system, which is composed of many kinds of immune cells, including cytotoxic lymphocytes, tumour infiltrating dendritic cells, regulatory T cells, tumour‐associated macrophages and myeloid‐derived suppressor cells, and tumour immune microenvironment is in dynamic change and may be different between different tumour types and individual patients.

The development of gene‐editing technology has facilitated and improved the manipulation of organoids at the gene level, making it possible to conduct genomic ‘batch’ research verified by biological behaviour. Combined with multi‐level and multi‐group research technology, organoid culture technology is an effective model for the study of breast cancer. It provides histological information on tumorigenesis and development, facilitates our understanding of the process of tumorigenesis and the driving factors of tumorigenesis and development, and allows exploration of new treatment models. Organoid culture technology can be used for high‐throughput screening of antitumour drugs. In addition, organoid culture maintains the original genotype and biological characteristics of the tumour, thereby facilitating the design of ultra‐precision individualized treatment strategies. Overall, organoid technology is the most intuitive and reliable model for individualized cancer research.

## CONFLICTS OF INTEREST

All authors declare that there is no conflict of interest.

## AUTHOR CONTRIBUTION

LPY and BEL performed the literature research and drafted the manuscript; HDC, RG, KHH, QYG, FL and WCC revised the manuscript, drafted the manuscript; LPY, BEL and JSH edited the final version and gave the final version and gave the final approval for the article to be published.
